# Large scale copy number variation (CNV) at 14q12 is associated with the presence of genomic abnormalities in neoplasia

**DOI:** 10.1186/1471-2164-7-138

**Published:** 2006-06-06

**Authors:** Ilan Braude, Bisera Vukovic, Mona Prasad, Paula Marrano, Stefanie Turley, Dwayne Barber, Maria Zielenska, Jeremy A Squire

**Affiliations:** 1 Department of Laboratory Medicine and Pathobiology, University of Toronto, Toronto, Ontario, Canada; 2 The Ontario Cancer Institute, Princess Margaret Hospital, Toronto, Ontario, Canada; 3 Department of Medical Biophysics, University of Toronto, Toronto, Ontario, Canada; 4 Department of Paediatric Laboratory Medicine, Hospital for Sick Children, Toronto, Ontario, Canada

## Abstract

**Background:**

Advances made in the area of microarray comparative genomic hybridization (aCGH) have enabled the interrogation of the entire genome at a previously unattainable resolution. This has lead to the discovery of a novel class of alternative entities called large-scale copy number variations (CNVs). These CNVs are often found in regions of closely linked sequence homology called duplicons that are thought to facilitate genomic rearrangements in some classes of neoplasia. Recently, it was proposed that duplicons located near the recurrent translocation break points on chromosomes 9 and 22 in chronic myeloid leukemia (CML) may facilitate this tumor-specific translocation. Furthermore, ~15–20% of CML patients also carry a microdeletion on the derivative 9 chromosome (der(9)) and these patients have a poor prognosis. It has been hypothesised that der(9) deletion patients have increased levels of chromosomal instability.

**Results:**

In this study aCGH was performed and identified a CNV (RP11-125A5, hereafter called CNV14q12) that was present as a genomic gain or loss in 10% of control DNA samples derived from cytogenetically normal individuals. CNV14q12 was the same clone identified by Iafrate *et al*. as a CNV. Real-time polymerase chain reaction (Q-PCR) was used to determine the relative frequency of this CNV in DNA from a series of 16 CML patients (both with and without a der(9) deletion) together with DNA derived from 36 paediatric solid tumors in comparison to the incidence of CNV in control DNA. CNV14q12 was present in ~50% of both tumor and CML DNA, but was found in 72% of CML bearing a der(9) microdeletion. Chi square analysis found a statistically significant difference (p ≤ 0.001) between the incidence of this CNV in cancer and normal DNA and a slightly increased incidence in CML with deletions in comparison to those CML without a detectable deletion.

**Conclusion:**

The increased incidence of CNV14q12 in tumor samples suggests that either acquired or inherited genomic variation of this new class of variation may be associated with onset or progression of neoplasia.

## Background

Genes typically affect an individual's susceptibility to a disease because mutations change either the amount or the composition of the protein encoded by the gene. It has recently been suggested that copy number variation may contribute to susceptibility to common diseases [1]. Microarray comparative genomic hybridization (aCGH) is a powerful tool that allows for high-resolution interrogation of genomic copy number imbalances of unique genomic sequences throughout the entire genome in a single experiment [2]. The repetitive fraction of the genome comprises diverse classes of repetitive DNA that is often polymorphic. Of these, the most recently discovered repeat-rich regions are those containing a high number of large-scale copy number variations (CNVs) [3] that involve the loss or gain of large fragments of DNA exceeding hundreds of kilobases. CNVs are present in the general population at varying degrees, and their role in cancer is poorly defined at the present time. While the mechanism responsible for generating the variation is unknown, CNVs have been shown to be preferentially located near known segmental duplications, or duplicons [4]. Duplicons are large regions of the genome (> 1 kb) that exhibit high sequence homology (90–98% sequence identity) [5], and have been linked to recurrent gene amplification in tumors [6].

Chronic myeloid leukemia (CML) is characterised cytogenetically by the Philadelphia translocation (t(9;22)), which results in the formation of a smaller than normal chromosome 22 known as the Philadelphia chromosome, and the derivative chromosome 9 (der(9)) [7]. While a great deal is known about the effects of the Philadelphia translocation, there is limited knowledge of the recombinational mechanisms that lead to genomic rearrangement. Saglio *et al*. first noted significant sequence homologies in the translocated regions and identified a 76 kb duplicon located near the *Abl1 *and *Bcr *genes on chromosomes 9 and 22 respectively. This duplicon may facilitate the Philadelphia translocation in CML by favouring homologous exchange between non-syntenic regions, or through double stranded breaks [8]. These exchanges suggest an error within a DNA repair mechanism responsible for maintaining genome integrity. Similarly, it has been suggested that defects of recombinational/repair pathways may facilitate acquisition of additional cytogenetic abnormalities in CML [9,10].

This study was initiated to investigate whether CNVs were associated with acquisition of more complex genomic alterations in CML. In particular, we were interested in determining if a CNV-dependent mechanism was associated with the presence of microdeletions seen on the der(9) in 15–20% of patients carrying a microdeletion [11,12]. The potential of CML patients with microdeletions of der(9) to be more generally associated with either acquired or inherited differences in CNVs has not been analyzed by aCGH methods. aCGH methods are beginning to document both the number of CNVs and their locations in phenotypically normal individuals [3,4]. It is not yet clear what role CNVs play in CML and cancer in general, however, it has been suggested that these sequences may be intrinsically unstable and may thus be hotspots for genomic alterations [3]. This study reports the identification of CNV14q12, a highly imbalanced region found to be present at a higher frequency in neoplastic samples in comparison to control DNA.

## Results

### Microarray comparative genomic hybridization (aCGH) analysis

**Figure 1:**
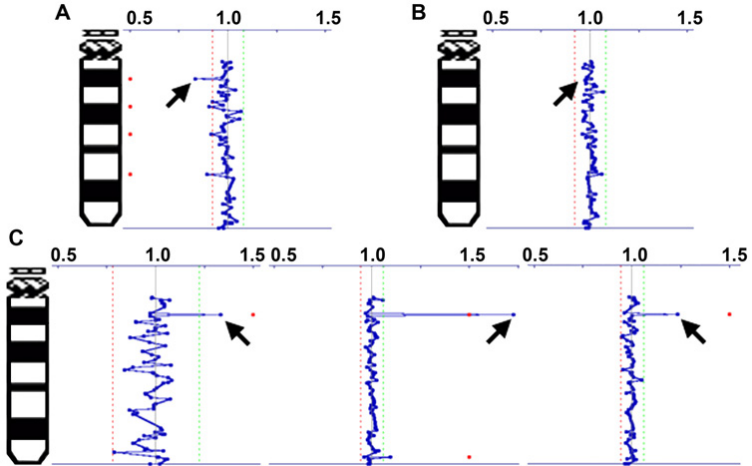
Microarray comparative genomic hybridization results for 5 chronic myeloid leukemia patients for chromosome 14. Arrow indicates CNV14q12. A) Arrow denotes 1 sample with a CNV14q12 deletion. B) Arrow denotes 1 sample with no change in the copy number of CNV14q12. C) Arrows show 3 samples with a copy number increase in the CNV14q12 region.

aCGH was performed on 5 chronic myeloid leukemia (CML) patients with a known deletion on the derivative 9 chromosome (der(9)). The presence of a deletion in these patients was assessed by real-time polymerase chain reaction (Q-PCR) and/or fluorescence in situ hybridization (FISH) [13]. aCGH was performed using commercially available 1–2 MB resolution Spectral Chip 2600 BAC arrays (Spectral Genomics, Houston TX, USA) and analyzed using *Normalise Suite *v2.5 [14]. An unexpected finding was the presence of a gain on chromosome 14 located at the BAC clone RP11-125A5, hereafter called CNV14q12, found in 3 of the 5 patient samples. The same clone showed a deletion in 1 CML sample (figure 1). The BAC clones on the array nearest to RP11-125A5 both upstream and downstream are RP11-529E4 and RP11-329K12, respectively, both of which showed normal copy numbers in all experiments, thereby defining the minimal region of gain to a 1.2 MB region centered at RP11-125A5.

**Figure 2:**
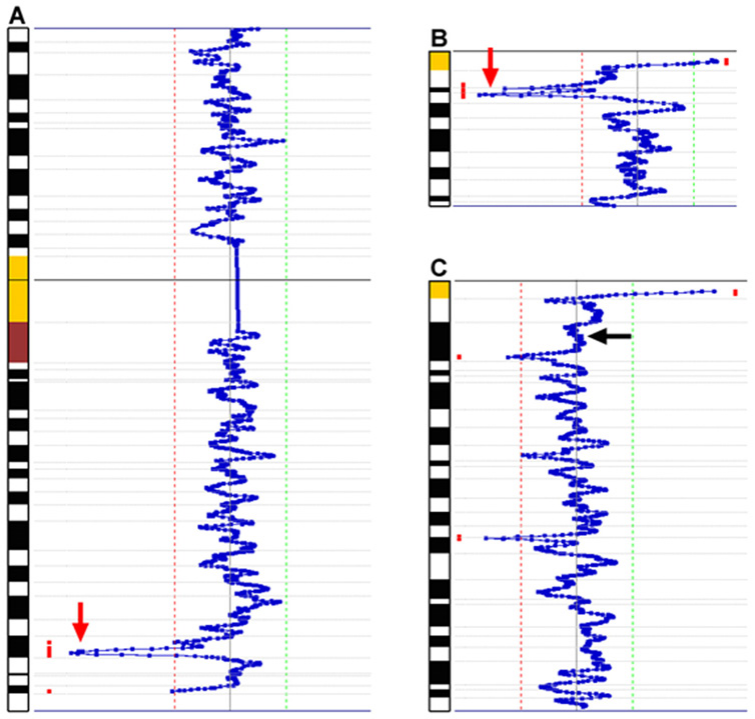
SMRT-array results showing the deletion on chromosomes 9 and 22 that are the result of the derivative 9 chromosome (der(9)) deletion in a chronic myeloid leukemia (CML) patient. The dotted red line denotes the 2 standard deviation cut-off to identify a deletion while the green dashed line marks the 2 standard deviation cut-off for a gain. A) Chromosome 9 alterations identified using the SMRT-array. The red arrow shows the deletion extending from 9q33.3 to 9q34.1. B) Chromosome 22. The deletion of chromosome 22 material, located around 22q11.2, on the der(9) chromosome is shown by the red arrow. C) Chromosome 14 from the same CML sample with the der(9) deletion, and shown to have the CNV14q12 event using another microarray comparative genomic hybridization platform as well as real-time polymerase chain reaction. The black arrow marks the site of CNV14q12 and, due to the use of a different normal genomic DNA sample, does not display the expected gain of genomic material. The most distal copy number variation was seen only in this sample and therefore not studied further.

aCGH analysis using a sub-megabase tiling path array (SMRT-array, BC Cancer Agency, Vancouver, Canada)[2], was performed on 2 samples with a deletion on the der(9) chromosome, as well as one sample not showing the der(9) deletion, all of which carry the CNV14q12 event (2 show a gain, and one a deletion). The gain or loss of genomic material at CNV14q12 does not correlate with the presence of a deletion on the der(9). Der(9) deletion status was determined by FISH analysis and confirmed by Q-PCR. Unfortunately, the normal control DNA used in these experiments was different from that used for both the 1–2 MB resolution microarrays, and the Q-PCR analysis, and it appears that this DNA carried the CNV14q12. As such, no other varitation was seen in any of the cases. The SMTR-array was able to identify the deletion on chromosomes 9 and 22 in one of the 2 cases where a deletion had been previously identified by FISH and Q-PCR analysis (figure 2). Project files for the Spectral Genomics and SMRT array dataset can be viewed [see Additional File 1] and [Additional File 2] respectively.

### Q-PCR results and statistical analysis

**Figure 3:**
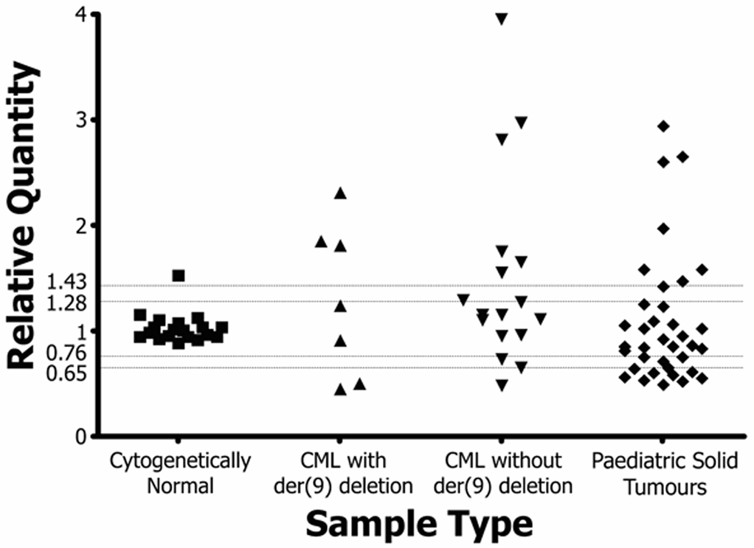
A graphical representation of real-time polymerase chain reaction results.

Relative quantification of the amount of genomic material at CNV14q12 was determined using Q-PCR, where the relative quantity is normalized to a haploid value. Based on all of the cytogenetically normal samples (excluding the single sample that showed a marked copy number gain) the mean haploid value was 1.02 with a standard deviation (SD) of 0.13. A 2 SD threshold was used to assign a normal copy range of 0.76 – 1.28. Based on Q-PCR analysis of 3 CML patient samples that had been shown to have a gain by aCGH analysis, the minimum value for a gain (mean – 2SD) was defined as 1.43 (1.99 ± 0.28). A deletion of CNV14q12 was found in one CML patient by aCGH analysis. The mean Q-PCR value for this sample was 0.45, establishing a range of 0.31–0.65 for a deletion. The results of the Q-PCR reactions based on the ranges defined here are summarized in table 1 (also see figure 3).

The chi-square test (χ^2^) was used to compare the copy number of the CNV14q12 variation (gains and losses) between each sample group. Based on χ^2 ^analysis, there was a statistically significant difference in the number of changes in cancer patients with additional cytogenetic abnormalities (both CML cohorts plus the paediatric solid tumor cohort) versus cytogenetically normal individuals (p ≤ 0.01). Individual analysis of CML patients with a deletion (p ≤ 0.001), CML without a der(9) deletion (p ≤ 0.01), and paediatric solid tumor samples (p ≤ 0.001) revealed a statistically significant difference between the number of variants in CNV14q12 when compared to cytogenetically normal individuals. Comparison of the number of variants in CNV14q12 seen in CML patients with and without a der(9) deletion showed a slight trend but no statistical difference in the frequency of the CNV14q12 variation in the presence of a der(9) deletion (p ≤ 1). However, the sample size of the CML patients with a deletion was small (n = 7) and thus statistical analysis was inappropriate. A larger patient cohort will be required to determine whether there is a statistical association present. Comparison of the CML cohorts, with or without a der(9) deletion, to the paediatric solid tumor cohort did not reveal a statistically significant difference (p ≤ 0.2 for both). As in the case of CML, a larger cohort of patients with a der(9) deletion may yet reveal a difference in the frequency of this variation as compared to paediatric solid tumors.

### Location of duplicated material using dual colour FISH

**Figure 4:**
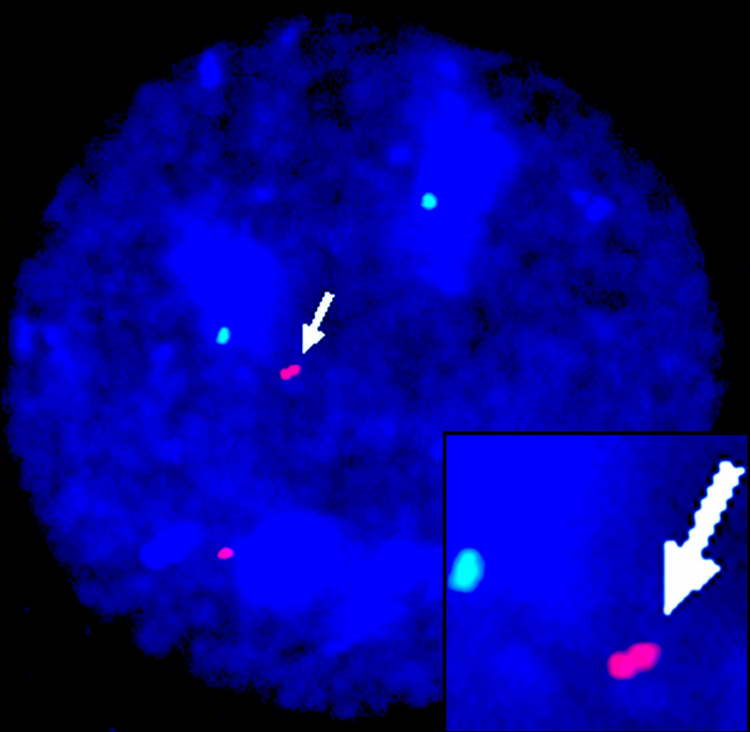
Fluorescence in situ hybridization (FISH) image of CNV14q12 (red) and RP11-79B13 (green), a control region on chromosome 14 that did not show a deviation from normal copy number according to the microarray comparative genomic hybridization experiments. The arrow marks the pair of FISH signals at CNV14q12. Quantitative-FISH showed that the marked probe was indeed due to hybridization at 2 adjacent loci rather than a split signal.

Dual colour FISH (D-FISH) was used to determine the location of the gained genomic DNA. The results (figure 4) identified a cytogenetic duplication of genetic material on a single chromosome in an adjacent position. Quantification-FISH (Q-FISH), a technique that utilizes the relative signal intensity of a target signal compared to a control probe [15], showed that there is a gain of CNV14q12 material [see Additional file 3].

### In silico analysis of the CNV14q12 genomic region

*In silico *analysis of the CNV14q12 genomic locus located on chromosome 14 in the q12 cytoband revealed the presence of 5 well characterised genes. Three of these genes are pseudogenes – BCL2/adenovirus E1B 19 kDa interacting protein (*BNIP3P*), ribosomal protein L26 pseudogene 3 (*RPL26P3*), and basic transcription factor 3 pseudogene 2 (*BTF3P2*) – while one is a hypothetical gene (*LOC387978*). The remaining gene is *FOXG1B *(forkhead box G1B; also called *BF1 *(brain factor 1), forkhead homolog-like 1 (*FKHL1*), *QIN *(QIN oncogene)). The role of *FOXG1B *has been extensively studied in neuronal development due to its expression in the human telencephalon. This gene is also expressed in the stomach, kidney, aorta, ovary and testis, and the cochlea. It is uncertain whether it is expressed in the bone marrow and whether *FOXG1B *would play a role in mediating survival in CML.

The genomic architecture of the CNV14q12 region includes 2 segmental duplications located upstream of RP11-529E4. Neither segmental duplication, nor any others, spans the amplified region in such a way that duplicons could facilitate the alterations observed here. L1-interspersed nuclear elements (LINEs) are found throughout the region which is not uncommon. Interestingly, 2 large LINE repeats (~6 kb and ~5.5 kb) are present at the 5' end of RP11-125A5. In addition, there are a large number of short interspersed nuclear elements that, like LINEs, are distributed throughout the genome.

## Discussion

Large-scale copy number variations (CNVs), and duplicons in general, are difficult to integrate into the human genome [5] and the high sequence homology has recently been shown to affect microarray comparative genomic hybridization (aCGH) results [16]. A study by Locke *et al*. showed that the presence of duplicon material in BACs spotted on a 15q11-q13 region specific array resulted in muted detection of genomic alterations. They also suggested that the presence of duplicons is a consideration when designing and analyzing microarrays for aCGH [16].

In a recent report on the presence of CNVs located in the genome of normal individuals, Iafrate *et al*. noted that 25% of the CNVs identified mapped to regions overlapping previously identified segmental duplications [4]. They showed that the correlation with known duplicons was significantly different from that determined for all clones on the array (p < 0.0001) [4]. Another report by Sebat *et al*. confirmed that CNVs were more prevalently located at known duplicon sites than would be expected if randomly distributed. Sebat *et al*. suggest that duplicons, unstable regions, and CNVs are probably the result of a common underlying mechanism [3].

The results presented in this study also suggest an increased incidence of the CNV14q12 imbalance in neoplasias such as chronic myeloid leukemia (CML) and solid tumors of childhood. Interestingly, in a study of 55 individuals, Iafrate *et al*. found similar results for CNV14q12 when examining cytogenetic aberrations in normal blood lymphocytes. [4] They performed aCGH analysis of 36 cytogenetically normal individuals as well as 16 individuals with a known constitutional cytogenetic abnormality. The results of this study identified a number of CNVs including 6 clones that were abnormal at a frequency of >20%. Among these clones was CNV14q12 (RP11-125A5). A chi-square comparison of the prevalence of individuals with and without additional cytogenetic abnormalities revealed a statistically significant increase in the likelihood of having a variation at this locus in the presence of additional cytogenetic abnormalities (p ≤ 0.001). This suggests that the mechanism underlying the formation of this CNV and the genomic abnormalities may be the same. However, because normal constitutional DNA was not available from any portrait within this study group, it was not possible to determine whether neoplastic cells acquired CNV as part of oncogenetic progression.

Fluorescence in situ hybridization (FISH) analysis of CML patient material revealed that CNV14q12 material was gained on a single chromosome located directly adjacent to the homologous material (figure 4). Since a split FISH signal would display the same pattern, quantification of the signal intensity over a minimum of 30 metaphases was performed on this CML patient sample as well as a series of cytogenetically normal individuals [see Additional file 3]. The results of this analysis revealed an increase in the CNV14q12 material thereby confirming the aCGH and the real-time polymerase chain reaction (Q-PCR) data. This duplication, along with the Q-PCR results that indicate both a gain and a loss to be present at this locus in the series of patients studied, suggests that duplication/deletion occur through incorrect homologous recombination between repeats flanking this genomic locus.

*In silico *analysis of the region surrounding RP11-125A5 failed to reveal any duplicons which would be a likely mechanism for mediating the CNV14q12 events, however, current genomic architecture of the CNV14q12 region is derived from sequence data obtained from a small number of individuals and it is possible that the frequency and diversity of CNV variation at 14q12 in humans may be much more extensive than is apparent by analysis using current NCBI datasets.

Analysis of L1-interspersed nuclear elements (LINEs) elements in the region did identify 2 large L1 elements of the PA7 subfamily. These elements are ~6 kb and ~5.5 kb respectively, and could possibly mediate unequal homologous recombination resulting in deletion or gain of a copy of this genomic region. LINE elements have been shown to play a role in disease formation through deletions of small (~5 kb) fragments of DNA [17]. A number of diseases have now been shown to be the result of rearrangements between repeat regions [17,18]. In general, the greater the distance between the repeats, the larger the repeats need to be in order to mediate the event [17]. A recent report has shown that LINEs [18] and palindromic AT-rich repeats are associated with acquired chromosome translocation frequencies that can mediate phenotypes generated by the duplication of a large fragment (~450 kb) of DNA [19].

## Conclusion

The results presented here identified a copy number variation, CNV14q12, which was observed at a high level in neoplastic DNA samples. The limitations of the sample size precluded a detailed statistical analysis of subsets within the study group. Despite being present at a high level in both chronic myeloid leukemia (CML) cohorts, the presence of CNV14q12 failed to show a strong significant association with the presence of the derivative chromosome 9 (der(9)) deletion. Likewise, the occurrence of the CNV14q12 variation was more prevalent in the paediatric tumor cohort than in control DNA. These data suggest that the occurrence of CNV14q12 may be indicative of an error in a pathway responsible for maintaining DNA integrity in neoplasia. A comparison of the cytogenetic profile of patients with CNV14q12 in a familial cohort would indicate whether this is an acquired event secondary to the primary cytogenetic abnormality, or a primary event indicative of faulty DNA replication pathways.

## Methods

### Samples

#### Cytogenetically normal samples

A series of 19 anonymous, ethnically diverse, cytogenetically normal samples were used for real-time polymerase chain reaction (Q-PCR) analysis. The samples were acquired according to the institutional guidelines of the research ethics board of the University Health Network Research Ethics Board (Toronto, Canada). Cytogenetic analysis of these samples failed to identify any abnormalities. All samples were derived from an unaffected parent as part of routine cytogenetic screening. Of these 19 control samples, 4 were of northern European origin, 3 from southern Europe, 1 Caucasian, 3 Jewish, 2 Native Indian, 2 Filipino, 2 Black, and 2 West Indian. In addition, one commercially available normal female genomic DNA sample was purchased from Promega (Napeen, Canada).

#### Chronic myeloid leukemia (CML) patient samples

Two groups of CML samples (derived from routine University Health Network laboratory diagnostic procedures according to the institutional guidelines of the research ethics board) were studied. The first group consisted of CML patients who, by Q-PCR and/or fluorescence in situ hybridization (FISH) analysis, were determined to carry a deletion on the derivative chromosome 9 (der(9)) at the site of the Philadelphia translocation breakpoint [13]. This group consisted of 7 patient samples. The second group, consisting of 17 patient samples, was identified as not having a deletion on the der(9) chromosome by Q-PCR analysis [13]. The two groups did not differ significantly in terms of age, percentage of blasts, or percentage of basophils. There was a difference in the ratio of males to females between the two groups, however this difference can be attributed to the small sample size of the der(9) deletion patients.

#### Paediatric solid tumor control cohort

Thirty-six tumor DNA samples derived from a broad selection of paediatric patients (12 medulloblastomas, 12 neuroblastomas, 7 Ewing's sarcomas, 5 Rhabdosarcomas) who were naïve to therapy were acquired according to the institutional guidelines of the research ethics board of the Hospital for Sick Children Research Ethics Board (Toronto, Canada). The samples were used as a control to determine the prevalence of an abnormal event at CNV14q12 in cancer samples.

#### Reference sample for Q-PCR analysis

The reference sample selected for these experiments was genomic male DNA purchased from Promega (Napeen, Canada). In addition, a genomic female DNA (Promega, Napeen, Canada) was also used for the Q-PCR analysis, after determining, based on Q-PCR analysis, that the relative copy numbers of both the male and female reference DNA were the same (p = 0.6572).

#### Microarray comparative genomic hybridization (aCGH) and microarray analysis

Genomic DNA was obtained from all tumor samples [see Additional file 4] using standard phenol chloroform extraction methods. The normal human reference DNA comprised of an equimolar mixture of DNA derived from multiple male donors (Promega, Madison, WI). The genomic array slides were obtained from Spectral Genomics (Houston, TX) and comprised of 2632 large insert clones (BACs/PACs) spaced ~1–2 Mb apart. The protocol used was as suggested by the suppliers. In brief, 2 μg each of genomic tumor and normal DNA were directly labelled with Cy3- or Cy5-dCTP (Amersham, Baie D'Urfe, Canada) using random priming. Following hybridization the microarrays were washed using 50% formamide/2 × SSC (20 minutes), 0.1% Ipegal/2 × SSC (20 minutes), and 0.2 × SSC (10 minutes) all pre-warmed to 50°C. A final wash with de-ionized distilled water was carried out. Air-dried microarray slides were scanned with an Axon GenePix 4000A confocal scanner, and fluorescence intensities quantified with the GenePix Pro 3.0 software (Axon Instruments, Union City, CA). Hybridizations were carried out in duplicate with fluor reversals to ensure that labelling differences did not affect imbalance assignments. Details concerning software, normalization, and imbalance assignments have been described previously [20] and are available at [21]. Using this software, a sliding window (n = 80 features) was used to remove some of the background noise present in the array, and a 2 standard deviation threshold was chosen for the assignment of imbalance.

Submegabase tiling path array (SMRT; BC Cancer Agency, Vancouver, Canada) used consisted of 32,433 bacterial artificial chromosomal DNA arranged in a tiling path spanning the entire genome. Hybridization was carried out as described by *Ishkanian et al *on 2 samples with a deletion of the der(9) chromosome, as well as one sample not showing the der(9) deletion, all of which carry the CNV14q12 variant. Normal human control DNA was derived from a pool of 6 normal individuals (Promega, Madison, WI), unfortunately this sample carried the CNV14q12 and as such no variation could be seen. This normal reference DNA was different from that used in the aCGH and Q-PCR analysis done on the Spectral Genomics Platform.

The data discussed in this publication have been deposited in NCBIs Gene Expression Omnibus (GEO) [22] and are accessible through GEO Series accession number GSE4860 [23].

#### Quantitative-fluorescence in situ hybridization (Q-FISH)

The probes used were selected using the Map Viewer program available from NCBI [24]. The BAC clones corresponding to regions of interest were obtained from BACPAC Resources Center operated by the Children's Hospital Oakland Research Institute [25]. The BAC clones selected were RP11-125A5 and RP11-79B13, both of which are located on chromosome 14. Clone RP11-79B13 was used as the control.

One microgram of the BAC clone was labelled overnight at 15°C using a Nick Translation kit (Vysis, Downers Grove IL, USA) with either spectrum green or spectrum orange. For each slide, 10 μl of probe was used. Each probe was initially verified by FISH mapping onto metaphase spreads generated from lymphocytes from a normal, healthy individual.

Image acquisition was performed using a Zeiss Axioskop 2 Plus microscope (Jena, Germany) equipped with the appropriate filter sets. As a normal control was performed with each experiment, the exposure time may have varied between experiments due to changes in the signal intensity; however, within each experiment the exposure time for each fluorochrome was identical so that quantification of the signal was applicable.

Analysis of the signal intensities was performed using ImageJ [26]. Q-FISH relies on a relative quantification of a signal of interest with respect to the intensity of the signal for a control region. A minimum of 30 individual nuclei were analyzed and the average ratio of the signal of interest to the control signal was compared to the ratio determined for the normal sample run in the same experiment. To confirm that the selected control clone was a suitable control for the analysis, a histogram of the control spot across the captured images was generated for each sample and compared to the histogram of the control signal in the normal sample. This comparison revealed highly similar single mode distribution of the control signal, thereby indicating that the control clone was suitable for the analysis (data not shown).

#### Q-PCR

The sequences used to design the primers to validate imbalance at CNV14q12 were obtained from end sequencing of the BAC clones RP11-125A5 (AQ345961) and RP11-79B13 (AQ284031). The selection of these clones was based on the analysis of the 5 Spectral Chip 2600 microarrays performed. aCGH analysis identified RP11-125A5 to be gained in 3 of the 5 cases and lost in 1 case, based on a threshold of 2 standard deviations (SD). Indeed the gain seen in CNV14q12 in all three cases was significant at up to 8 SD as was the loss. RP11-79B13 was selected as the control clone, as in none of the microarrays was this region altered, with the deviation from baseline at this clone being negligible (normalized values of 0.988–1.007, SD = 0.009, for all 5 arrays). The control region selected was also on chromosome 14 in order to negate the impact of ploidy change of this chromosome on the Q-PCR analysis.

Primers for the study (table 2) were designed using primer3 [27]. BLAST analysis [28] showed the sequences to be specific for the region of interest. Q-PCR reactions were carried out as previously described [13]. Briefly, each reaction included the optimal primer concentration (table 2), 20 ng DNA, 10 μl of 2X SYBR Green master mix (Applied Biosystems, Foster City, CA), in a volume of 20 μl. Cycling conditions began with 5 minutes at 50°C followed by 10 minutes at 95°C. This was followed by 40 cycles of: 15 seconds at 95°C, 1 minute at 60°C. Q-PCR was carried out on an ABI Prism 7900HT instrument with a 384-well block (Applied Biosystems, Foster City, CA). All reactions were performed in triplicate. A sample was considered to have a failed reaction and need repeating in the event that more than one of the triplicates failed. Relative quantification using the ΔΔC_T _method with a threshold of 0.2 was used for the analysis of the Q-PCR results.

## Authors' contributions

IB participated in the design of the study; carried out the genomic studies and performed the statistical analysis; and drafted the manuscript.

BV assisted in quantitative-FISH and statistical analysis.

MP participated in preparation, editing and submission of the manuscript.

PM participated in preparation, editing and submission of the manuscript.

ST involved in acquisition and coordination of clinical sample for this study.

DB participated in the design of the study and editing of the manuscript.

MZ acquisition and coordination of clinical sample for this study, participated in the design of the study and editing of the manuscript.

JS conceived of the study, participated in its design and coordination and manuscript preparation and editing.

All authors read and approved the final manuscript

## Supplementary Material

Additional file 1 Supplementary data Figure A. shows the Quantitative-FISH results from 2 CML patients. Click here for file

Additional file 2 List of samples used on Spectral Genomics and SMRT arrays. Click here for file

Additional file 3 Project files for Spectral Genomics dataset, can be viewed by MS Excel or Normalise Suite v2.5. Click here for file

Additional file 4 Project files for SMRT array dataset, can be viewed by MS Excel or Normalise Suite v2.5. Click here for file

## Figures and Tables

**Table 1:** Real-time polymerase chain reaction results. Occurrence of a copy number variation at 14q12

Cohort	Change	No Change	Total	χ^2 ^p-value
Normal	2	23	25	
CML* with der(9)^+ ^deletion	5	2	7	≤ 0.001
CML no der(9) deletion	8	9	17	≤ 0.01
All CML	13	11	24	≤ 0.001
Paediatric Solid Tumor	21	22	43	≤ 0.001
All Tumors	34	33	67	≤ 0.001

*Chronic myeloid leukemia

^+^derivative 9 chromosome

**Table 2:** Primer information. Primers used to assess the CNV14q12 and RP11-79B13 genomic regions.

Primer	Accession	Sequence	Size	Conc.
125A5_F	AQ345961	AGCATGTCCCTCTCTCAACC	118 bp	50 nM
125A5_R	AQ345961	GCAGCCTATAACCCCCTTTT		
79B13_F	AQ284031	CAGGATGTCCACAGCCATAA	140 bp	100 nM
79B13_R	AQ284031	TCTGTTGGGTCCTGAGGAAG		
