# Supplementary material for: Large scale copy number variation (CNV) at 14q12 is associated with the presence of genomic abnormalities in neoplasia
Source: BMC Genomics. 2006 Jun 6;7:138. doi: 10.1186/1471-2164-7-138 (PMC1550726; doi:10.1186/1471-2164-7-138)
Supplement: Additional file 3 — Project files for Spectral Genomics dataset, can be viewed by MS Excel or Normalise Suite v2.5. [file 1471-2164-7-138-S3.doc]

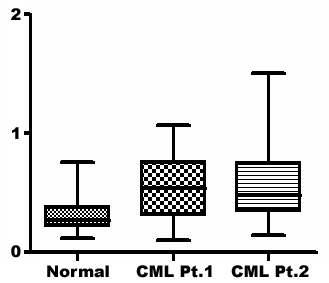


Additional file 3: Quantitative-FISH results from 2 CML patients and one cytogenetically normal control sample. The median relative signal intensity ratio derived from a minimum of 30 interphase nuclei, as well as the range of the signals from each of the cells analysed, is shown. The student’s t-test was used to statistically analyze the samples and the p-values for this analysis are shown.
